# Gastric Antral Vascular Ectasia as an Uncommon Cause of Upper Gastrointestinal Bleeding: A Case Report

**DOI:** 10.7759/cureus.110600

**Published:** 2026-06-10

**Authors:** Rakin Muhtadi, Kenneth D Katz

**Affiliations:** 1 Department of Emergency and Hospital Medicine, Jefferson Health - Lehigh Valley Region, Bethlehem, USA; 2 Division of Medical Toxicology, Department of Emergency and Hospital Medicine, Jefferson Health - Lehigh Valley Region, Bethlehem, USA; 3 Morsani College of Medicine, University of South Florida, Lehigh Valley Campus, Allentown, USA

**Keywords:** gave, gi bleed, stomach vascular lesions, upper gastrointestinal bleeding, watermelon stomach

## Abstract

Gastric antral vascular ectasia (GAVE) can result in upper gastrointestinal bleeding (UGIB) and is an important diagnostic consideration, especially in the elderly. It is often mistaken for portal hypertensive gastropathy given its similar endoscopic appearances and occurrence in comparable patient populations. We present the case of an elderly patient who presented to the emergency department (ED) with UGIB due to GAVE.

A 75-year-old female patient with a past medical history of peptic ulcer disease, hypertension, rheumatoid arthritis, and type II diabetes mellitus presented to the ED for repeat syncopal episodes, nausea, diarrhea, and fatigue. The patient had melanotic stool, significant blood-loss anemia, and an abdominal computed tomography (CT) scan showing hepatic cirrhosis. The patient was administered intravenous packed red blood cells, ceftriaxone, octreotide, and pantoprazole, and was admitted to the hospital. Esophagogastroduodenoscopy (EGD) showed no varices but GAVE, which was then treated with argon plasma coagulation. The patient experienced no further bleeding and was discharged on hospital day 3.

GAVE is a rare cause of UGIB with which emergency physicians should be familiar. Immediate stabilization in the ED, followed by prompt and direct endoscopic therapy, is critical for its treatment.

## Introduction

Gastric antral vascular ectasia (GAVE), while uncommon, can result in significant upper gastrointestinal bleeding (UGIB). In one study, it represented around 4% of nonvariceal UGIB [1]. Its pathophysiology is unknown, but it is associated with a myriad of comorbid conditions. An estimated 30% of patients with GAVE have concomitant liver disease, whereas GAVE is observed in approximately 1 in 40 patients with end-stage liver disease [2]. Although large-scale prevalence data are limited, one cohort study of 45 patients reported autoimmune connective tissue disorders in 62%, with the most common being Raynaud’s phenomenon (31%) [3]. Additionally, GAVE has been associated with hypertension and chronic kidney disease [4].

Since both GAVE and portal hypertensive gastropathy (PHG) can cause UGIB in cirrhotic patients, early esophagogastroduodenoscopy (EGD) is essential to differentiate between the two. Unlike PHG, GAVE does not respond to beta-blockers or transjugular intrahepatic portosystemic shunt (TIPS) procedure [5]. One key difference is that GAVE is usually limited to the antrum, while PHG is predominant in the fundus and corpus [5,6]. We present the case of a patient who presented to the emergency department (ED) with UGIB and was ultimately diagnosed with GAVE.

## Case presentation

A 75-year-old female patient with a past medical history of peptic ulcer disease, hypertension, rheumatoid arthritis, and a remote history of blood transfusion for melena decades prior presented to the ED with rectal bleeding and syncope. Other notable medical history included type II diabetes mellitus, hypothyroidism, osteoarthritis, and latent tuberculosis infection. She was prescribed omeprazole, amlodipine, folic acid, metformin, methotrexate, and hydrochlorothiazide. She stated that she had experienced approximately five syncopal episodes and bloody diarrhea for two weeks prior to ED presentation. The patient also described dark stool with accompanying lower abdominal pain starting two days before ED presentation. On the day of presentation to the ED, she experienced another syncopal episode. Prior to arrival, she also had several episodes of non-bloody, non-bilious vomiting and multiple dark stools. She denied any tobacco, alcohol, or nonsteroidal anti-inflammatory (NSAID) medication use.

In the ED, her vital signs were as follows: blood pressure 118/61 mmHg, pulse 82 bpm, respiratory rate 16 rpm, and oxygen saturation of 100% on room air. Her physical examination was significant for left-sided abdominal tenderness and gross melena. Her electrocardiogram was unremarkable. The most notable laboratory abnormality was a markedly low hemoglobin level (6.7 g/dL, Table 1). Her last measured hemoglobin was 13.7 g/dL five months prior to ED presentation.

**Table 1:** Laboratory test results drawn at the patient’s initial presentation to the emergency department.

Test	Results	Reference range
Hemoglobin	6.7 g/dL	11.5-14.5 g/dL
White blood cells	5.5 thou/cmm	4-10 thou/cmm
Blood urea nitrogen (BUN)	24 mg/dL	7-25 mg/dL
Creatinine	0.99 mg/dL	0.4-1.10 mg/dL
Lipase	105 U/L	11-82 U/L

Because gross melena was detected on physical examination, and the patient had very low hemoglobin, the patient received 1 unit of packed red blood cells. Additionally, a CT of the abdomen and pelvis was ordered due to the patient’s abdominal pain and to identify any potential etiology of gastrointestinal bleeding. The CT demonstrated possible hepatic cirrhosis, distal paraesophageal varices, and colonic diverticulosis (Figure 1).

**Figure 1:**
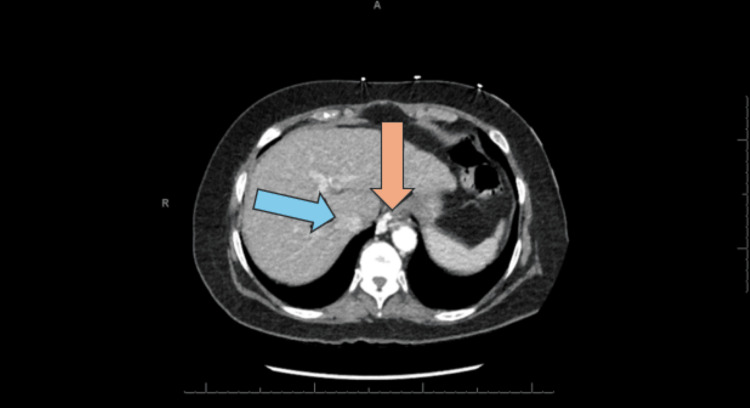
Axial image from CT of the abdomen and pelvis demonstrating dilated paraesophageal vessels (orange arrow) and liver parenchymal heterogeneity consistent with cirrhosis (blue arrow).

Despite the abdominal pain and mild elevation in lipase level, this elevation was not more than three times the upper limit of normal, and coupled with no CT findings of pancreatitis, this diagnosis was excluded. With the significant decline in hemoglobin, but preserved resting hemodynamics, it was likely that she had been experiencing blood loss over a protracted period of time. Her repeat episodes of syncope in the weeks prior to presentation support this as well. However, due to the CT findings of cirrhosis and varices, the patient was administered intravenous (IV) ceftriaxone 2000 mg and IV octreotide 50 mcg and admitted to the hospital.

On hospital day (HD) 1, the patient had a gastroenterology evaluation and subsequently underwent EGD, which showed no varices but instead linear erythematous lesions (Figure 2). Argon plasma coagulation was applied to the area around the pylorus that was mildly bleeding. Her EGD findings were consistent with GAVE. The patient was maintained on oral omeprazole 40 mg daily. On HD 2, her hemoglobin remained stable without any further melena, and she was discharged on HD 3 with gastroenterology follow-up.

**Figure 2:**
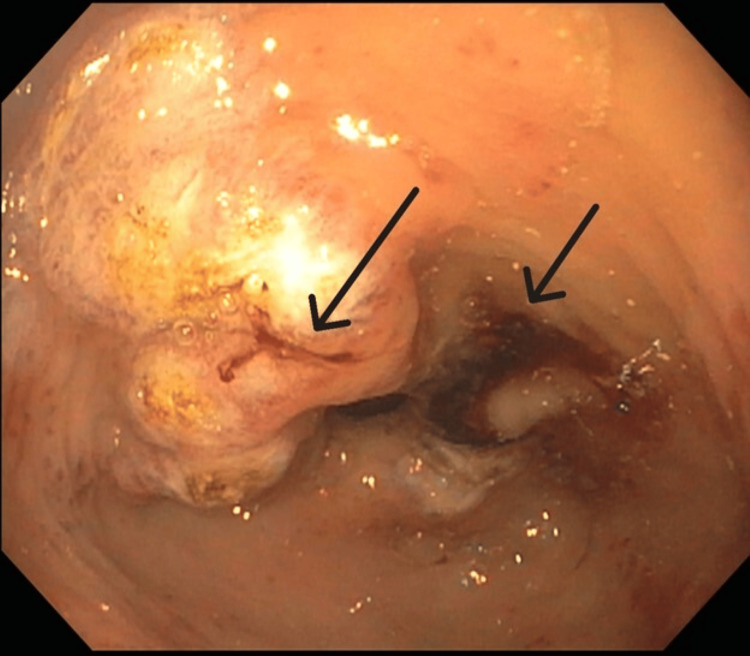
Linear erythematous lesions (arrows) in the gastric antrum consistent with gastric antral vascular ectasia.

## Discussion

The patient’s clinical presentation and initial laboratory and CT findings were concerning for cirrhosis with accompanying variceal bleeding, thus prompting administration of IV ceftriaxone and octreotide. PHG and GAVE were also included in the differential of causes of UGIB in patients with cirrhosis. Both peptic ulcer disease and gastritis, causing abdominal pain and UGIB, were also considered but less likely given the consistent use of omeprazole and lack of alcohol, tobacco, or NSAID use. However, the patient was maintained on IV pantoprazole until EGD was performed. With the report of the patient having bright red and then dark stool, we considered a UGIB, which was initially brisk but achieved some degree of clot formation, versus a mixed UGIB-LGIB picture. Considering that the patient had been mostly reporting melena for the last 24 hours prior to presentation, we suspected a UGIB to be more likely.

The patient described in this case report had multiple risk factors for GAVE. She had hypertension and rheumatoid arthritis and, given the CT findings, had newly developed cirrhosis; all of these medical conditions are associated with GAVE [1,3]. Since its recognition and naming by Jabbari et al. in 1984, large, multicenter epidemiologic data on GAVE have been lacking [7]. A review of 164 GAVE patients found that 88% presented with iron deficiency anemia, predominantly occult gastrointestinal bleeding, with only 15% presenting with melena [8]. Gostout et al. presented a large case series from a single center of 45 patients, reporting that 89% had only occult bleeding and 62% needed blood transfusions in the 12 months prior to treatment for GAVE [3]. Furthermore, GAVE is more common in older women. Gostout et al. found that 71% of their cohort with GAVE were women with a mean age of 73 years [3]. The patient presented in this case report represents a severe manifestation of GAVE, with marked melena and anemia. However, considering she was experiencing syncope for two weeks prior to her presentation and her compensated hemodynamics, it is likely that she had been having occult gastrointestinal bleeding for weeks, which fits with the typical GAVE presentation. Importantly, the most critical action performed in our patient was the urgent EGD in the hospital, which led to a rapid diagnosis and argon plasma coagulation.

The pathophysiology of GAVE remains unclear. Research is limited to case studies with small patient populations and is often focused on the subset of patients who have GAVE in the setting of cirrhosis. Quintero et al. found that patients with GAVE and cirrhosis had elevated levels of gastrin and low levels of pepsinogen [9]. Gastrin, produced by G cells in the gastric antrum, has a vasodilatory effect; however, it remains unclear whether this vasodilation ultimately leads to UGIB in GAVE [10]. Another theory is repeated mechanical trauma. Patients with GAVE and cirrhosis have impaired antral motility [11]. Gastric mucosa could be drawn through the pyloric ring, leading to repeated shear forces that cause fibromuscular hyperplasia and ectatic vessels.

The diagnosis and primary treatment of GAVE are both via EGD. Endoscopic plasma coagulation controls bleeding in most patients, and endoscopic band ligation is emerging as an alternative approach [3,12]. Antrectomy is an option for patients with recurrent bleeds who fail endoscopic therapies. While PHG can appear similarly to GAVE during EGD with erythematous streaks on the gastric mucosa, the patterns in PHG are usually polygonal but linear in GAVE [13]. Despite their similar appearance, neither reducing portal pressures with beta-blocker medications nor performing a TIPS procedure controls UGIB in patients with GAVE [14].

## Conclusions

Our patient case represents a severe presentation of GAVE, a rare cause of UGIB but with potential to cause significant morbidity, often progressing to transfusion-dependent anemia. Emergency medicine physicians should be familiar with GAVE so that they can facilitate prompt EGD for both diagnosis and treatment, especially since GAVE has clinical characteristics similar to those of variceal bleeding and PHG, both of which require different therapeutics. Suspicion for a possible diagnosis of GAVE should be high, especially in older women presenting with concern for occult UGIB, iron deficiency anemia, and comorbid liver or autoimmune disease.
